# Critical evaluation of arguments opposing male circumcision: A systematic review

**DOI:** 10.1111/jebm.12361

**Published:** 2019-09-08

**Authors:** Brian J Morris, Stephen Moreton, John N Krieger

**Affiliations:** ^1^ School of Medical Sciences University of Sydney Sydney New South Wales Australia; ^2^ CircFacts Warrington England UK; ^3^ Department of Urology University of Washington School of Medicine Seattle Washington

**Keywords:** complications, public health policy, sexual function, sexually transmitted infection, urinary tract infection

## Abstract

**Objective:**

To systematically evaluate evidence against male circumcision (MC).

**Methods:**

We searched PubMed, Google Scholar, EMBASE and Cochrane databases.

**Results:**

Database searches retrieved 297 publications for inclusion. Bibliographies of these yielded 101 more. After evaluation we found: Claims that MC carries high risk were contradicted by low frequency of adverse events that were virtually all minor and easily treated with complete resolution. Claims that MC causes psychological harm were contradicted by studies finding no such harm. Claims that MC impairs sexual function and pleasure were contradicted by high‐quality studies finding no adverse effect. Claims disputing the medical benefits of MC were contradicted by a large body of high‐quality evidence indicating protection against a wide range of infections, dermatological conditions, and genital cancers in males and the female sexual partners of men. Risk‐benefit analyses reported that benefits exceed risks by 100‐200 to 1. To maximize benefits and minimize risks, the evidence supported early infant MC rather than arguments that the procedure should be delayed until males are old enough to decide for themselves. Claims that MC of minors is unethical were contradicted by balanced evaluations of ethical issues supporting the rights of children to be provided with low‐risk, high‐benefit interventions such as MC for better health. Expert evaluations of case‐law supported the legality of MC of minors. Other data demonstrated that early infant MC is cost‐saving to health systems.

**Conclusions:**

Arguments opposing MC are supported mostly by low‐quality evidence and opinion, and are contradicted by strong scientific evidence.

“The human understanding when it has once adopted an opinion (either as being the received opinion or as being agreeable to itself) draws all things else to support and agree with it. And though there be a greater number and weight of instances to be found on the other side, yet these it either neglects and despises, or else by some distinction sets aside and rejects, in order that by this great and pernicious predetermination the authority of its former conclusions may remain inviolate.”

*Sir Francis Bacon*, The New Organon, *1620*.

## 1 INTRODUCTION

Compelling data, such as randomized controlled trials (RCT), systematic reviews and meta‐analyses, showing net benefits of male circumcision (MC) to males and their female sexual partners led the American Academy of Pediatrics (AAP) in 2012^1^, ^2^ and the US Centers for Disease Control and Prevention (CDC) in 2018^3^ to release affirmative guidelines in support of nontherapeutic early infant MC (EIMC) and nontherapeutic MC of older males. These statements supersede older policies in the United States, as well as nonevidence‐based negative policies in other countries^4^, ^5^, ^6^, ^7^ (Table 1).

**Table 1:** Organizations opposed to nontherapeutic MC of boys

**Non‐US medical bodies having formal policy statements** British Medical Association (2006)^4^ Royal Australasian College of Physicians—Paediatrics & Child Health Division (2010)*, 5 Royal Dutch Medical Association (KNMG) (2010)^6^ Canadian Pediatric Society (2015)**, 7
**Small quasi‐professional organizations** Doctors Opposing Circumcision (DOC) Attorneys for the Rights of the Child (ARC)
**Lay lobby groups** National Organization of Circumcision Information Research Centers (NOCIRC) National Organization to Halt the Abuse and Routine Mutilation of Males (NOHARMM) National Organization for Restoring Men (NORM) International Coalition for Genital Integrity Intact America Bloodstained Men (BSM) Mothers Against Circumcision The VMMC Experience Project***

^*^Policy is currently in the process of being updated.

^**^Only recommends nontherapeutic MC for “*boys in high‐risk populations and circumstances*.”

^***^Opposition by this group is directed at MC irrespective of age, with a particular focus on the voluntary medical male circumcision (VMMC) programs currently underway in sub‐Saharan Africa.

Various individuals, certain small professional organizations and lay lobby groups (Table 1) actively discourage nontherapeutic circumcision of boys. Members adopt various tactics, including the use of social media, to influence parents, physicians, academics and others regarding MC.^8^, ^9^, ^10^, ^11^, ^12^ Contradicting the AAP and CDC policy recommendations, opponents have lobbied to have MC of minors banned in the United States^13^ and Scandinavian countries, although to date such efforts have not been successful.^14^, ^15^, ^16^, ^17^ Arguments opposing nontherapeutic MC, especially in minors, appear to start with the premise that MC has no benefits, only harms, or that any benefits only apply later in life when the male can make his own decision to get circumcised.^18^, ^19^, ^20^, ^21^ In this “posttruth” era, vocal minority groups consider that their opinions count more than those of medical and scientific experts.^22^ These attitudes fit with a pattern of radical individualism, devaluation of scientific evidence, and promotion of autonomy, in which life‐saving childhood vaccines, for example, may be refused by parents, as is their legal right, which must be respected, except when parents are not in agreement.

To help provide clarity to this vexing issue, especially given the adverse consequences to global public health and individual well‐being of getting MC policy wrong, the aim of the present systematic review was to evaluate the arguments made against nontherapeutic MC (summarized in Table 2), as well as assertions by MC opponents of purported functions of the foreskin that are lost to circumcision (listed in Table 3). In particular, we examine the extent to which arguments used to oppose nontherapeutic MC are supported by current scientific evidence. In our article, benefits (and harms) of nontherapeutic MC (hereinafter referred to simply as “MC” and “EIMC”) are judged according to the difference in prevalence of an adverse medical condition in those who have received MC compared with those who have not.

**Table 2:** Common arguments used in opposing nontherapeutic MC of minors

MC for prevention of urinary tract infections in infancy is unnecessary as these are rare, of minor consequence, and easily treated with oral antibiotics MC causes physical harm, including a high rate of surgical complications, numerous deaths, disrupts breastfeeding, commonly results in meatal stenosis and glans keratinization MC “pain” can result in permanent brain damage, autism, alexithymia, and post‐traumatic stess disorder MC reduces sexual function in men MC reduces sexual pleasure in men and their female sexual partners MC does not protect against infection with HIV or other sexually transmitted infections during heterosexual intercourse with an infected partner Condoms afford complete protection against HIV and other STIs, so obviating the need for MC MC is not needed for prevention of phimosis and penile inflammatory conditions since these can be easily treated with steroid creams Penile cancer is so rare that prevention by MC is not worth the effort MC should be delayed until the boy is old enough to make the decision for himself Non‐therapeutic MC of minors should be deemed unethical and illegal Early infant MC is a waste of money

**Table 3:** The “16 Functions of the Foreskin” argument^152^

Erotic pleasure especially via the ridged band and 20 000 nerve endings (Meissner's corpuscles) Acts as a rolling bearing in intercourse and masturbation Prevents dyspareunia (painful intercourse) Simulates partner's genitalia, giving her erotic pleasure Supplies skin to cover the shaft in erection and prevent tightness Stores pheromones and releases them on arousal Stores, releases and helps distribute natural lubricants (“smegma” and preejaculatory fluid) Makes the glans a visible signal of sexual arousal Provides a seal against the vaginal wall to contain semen Prevents the glans becoming keratinized, and keeps it soft and moist Protects the thin‐skinned glans against injury Protects the nerves of the glans and their erotic function In infancy, it protects the urethra against contamination, UTIs and meatal stenosis Provides lysosomes for bacteriostatic action around the glans Pigmented, it protects the unpigmented glans against sunburn Being vascular (rich in blood vessels that bring heat to the tissues), it protects the less vascular glans against frostbite and other weather‐related conditions

## 2 METHODS

### 2.1 Literature searches

**Figure 1:**
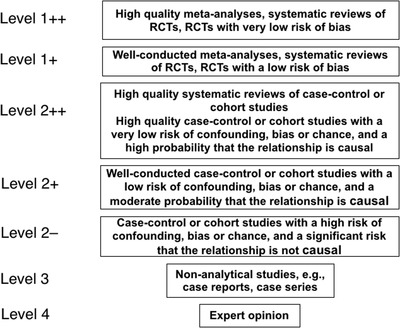
The hierarchy of quality of evidence used in science to evaluate claims, as specified by the Scottish Intercollegiate Guidelines Network (SIGN)^23^

We conducted sequential literature searches of PubMed, Google Scholar, EMBASE, and the Cochrane Systematic Review database for articles dating from 1 January 2005 until 31 December 2018. PubMed searches used the keyword “circumcision” in combination with one of 35 other relevant keywords shown in Supporting Information. An extraction file was created for each set and examined by the authors. Google Scholar, EMBASE, and Cochrane database searches to find additional references used “circumcision” as keyword. An update of the PubMed search was performed on 31 March 2019. Bibliographies of articles were examined to retrieve further key references. Inclusion criteria were publications arguing against MC, critiques of those publications, and other key publications. In accord with the hierarchy of scientific evidence, articles were graded for quality using the Scottish Intercollegiate Guidelines Network (SIGN) grading system^23^ (Figure 1). In instances in which a MC‐related topic had been the subject of recent high‐quality systematic reviews or meta‐analyses (level 1++ or 1+ evidence), these were cited for efficiency instead of all the individual studies on that topic. Internet searches were conducted for other relevant information, including MC policies. The study complied with Preferred Reporting Items for Systematic Reviews and Meta‐Analyses (PRISMA).^24^

## 3 RESULTS AND DISCUSSION

### 3.1 Articles retrieved and included

**Figure 2:**
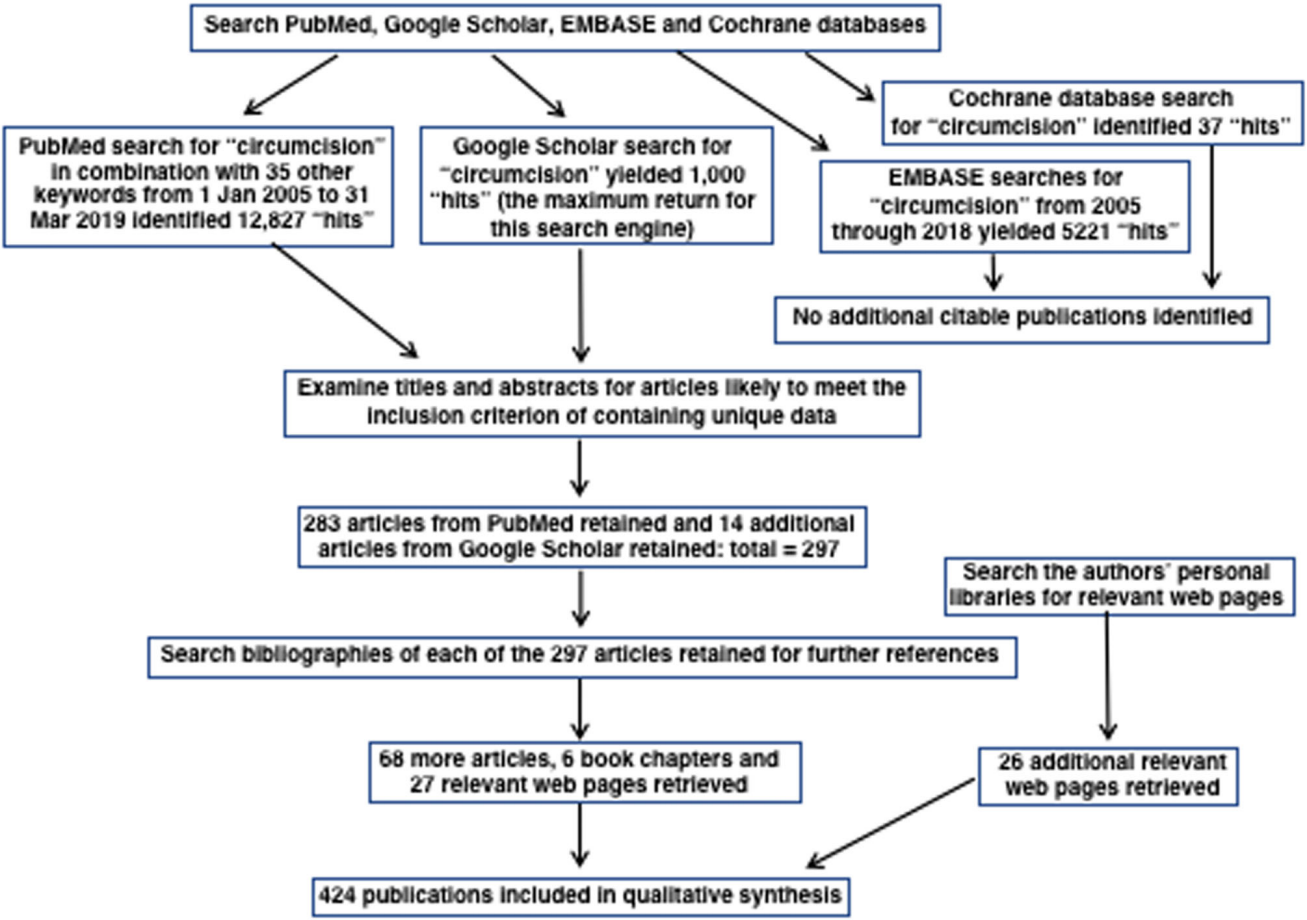
Search strategy diagram as required by PRISMA guidelines^24^

PubMed searches for 2005 through 2018 yielded 12 754 “hits” (Supporting Information), with 73 more “hits” to 31 March 2019. From these we identified 283 publications that met the inclusion criteria. A Google Scholar search yielded 14 additional articles from the maximum return of 1000 “hits” for this search engine. Searches of the EMBASE database and Cochrane Central Register of RCTs yielded, respectively, 5221 and 37 “hits,” but did not generate additional citable articles. In total, database searches yielded 297 articles for inclusion. Examination of bibliographies of the articles chosen yielded 68 further articles and 6 book chapters. Thus, total number of articles and book chapters included was 364. Bibliography searches also identified 27 relevant web pages for inclusion, and 26 more were identified by searches of the authors’ personal libraries. Figure 2 summarizes the search strategy in accord with the PRISMA statement.^24^ Two “in press” articles by the first author were also included. In order to address a comment by one of the reviewers, four publications on vaccination were included.

### 3.2 Urinary tract infection

The most recent meta‐analysis reported UTI incidence as 10 times lower in circumcised versus uncircumcised infant males.^25^ Cumulative incidence was 0.1% versus 1%, respectively. Infant UTI has been regarded by some as rare,^19^, ^26^ although pediatric urologists consider it to be a common problem.^27^ Up to 2 years of age, UTI rate was 0.59 versus 2.68 per 100 person‐years, respectively (*P* < .0005) and number needed to treat (NNT) with MC was reported as 39, decreasing to 29 when other sequelae were included.^28^ (For comparison, influenza vaccination of 50 children can prevent one outpatient visit (NNT = 50).^29^) UTI in infancy can result in significant morbidity^30^ and is the most common cause of sepsis in male neonates.^31^ Within the first 2 years incidence of pyelonephritis (0 vs 0.67), kidney disease (0.063 vs 0.13), hypertension (0.031 vs 0.27), and vesicoureteral reflux (0.13 vs 0.27) per 100 person‐years was lower in 2334 neonatally circumcised versus 573 uncircumcised US infant males, respectively.^28^ Rate for all diagnoses combined was 0.65 versus 3.5 (*P* < .0001).^28^ Progression to renal damage occurred in 19% of children with UTI aged < 2 years.^32^

Studies questioning the value of EIMC for UTI prevention^33^, ^34^ contained flaws,^25^, ^35^, ^36^ as we will explain. Sample collection and UTI diagnosis is more challenging in infant males than in older children or adults. Patients are more likely to present with nonspecific systemic symptoms, and history must be obtained from the parents. Of infant males with UTI, 27.6% were hospitalized in a US study, so adding to costs.^37^

Arguments that infants with UTI can be easily treated with oral antibiotics^19^, ^26^ apply to older males as oral administration in infants is difficult and absorption is low, requiring hospitalization to enable intravenous antibiotic administration.^14^, ^38^ Emergence of resistance to most or all antibiotics, including methicillin, will make treatment of UTI more challenging.^39^, ^40^, ^41^, ^42^, ^43^, ^44^ Maternal antibiotic use during pregnancy also increase the risk of resistant pathogens during neonatal UTI.^45^ Subpreputial swabs of boys aged 7 days to 11 years identified 50 bacterial isolates, most being multidrug‐resistant strains,^46^ and of boys aged 2 months to 9 years identified 72 microorganisms, including 54 Gram‐positive bacteria (57% enterococcus species), 17 Gram‐negative bacteria (41% *Escherichia coli*) and Candida species.^47^ Of swabs from healthy males (mean age 26.5 years), 17% from uncircumcised contained potentially uropathogenic Gram‐negative rods compared with 4% from circumcised males, and Streptococci, strict anaerobes, and genital mycoplasmas were only present in the uncircumcised.^48^ A RCT found that MC significantly reduced both the prevalence and load of genital anaerobic bacteria.^49^

A meta‐analysis found lifetime cumulative incidence of UTI in uncircumcised males was 32.1% and in circumcised males was 8.8%.^25^ Number needed to treat was 4.29,^25^ with the foreskin contributing to 72.6% of lifetime UTI risk in an uncircumcised male UTI patient.

In summary, EIMC reduces the substantial risk of UTI in infancy and beyond.

## 4 PHYSICAL HARM

### 4.1 Terminology

MC has been termed, “*male genital mutilation*,”^13^ a term adopted from *“female genital mutilation,”* which has no medical benefits and is often harmful. Mutilation means damage or disfigurement. Below we examine whether this applies to MC. MC has also been referred to as *“amputation,”* a term used in the medical literature when referring to removal of a limb, digit, or the entire penis. A belief in physical harm underlies arguments that MC presents, “*intractable moral, child abuse, human rights, and ethical problems*,”^50^ the veracity of which will be addressed in the section on ethical issues.

### 4.2 Immediate complications of MC and their frequency

A 2014 study by CDC researchers of 1.4 million circumcisions in the United States, based on inpatient data as well as data from more than 870 000 unique outpatient medical providers, found frequency of adverse events associated with EIMC was 0.4%.^51^ Adverse events were 20 times more frequent in boys aged 1‐9 years, and were 10 times higher for males aged ≥10 years in this study. Adverse events were 0.5% in neonates and 18.5 times higher in nonneonates in a recent large California study.^52^ The AAP's 2012 policy statement^1^ reported figures of 0.19%^53^ and 0.22%^31^ from two large US studies, and 0.34% from a large Israeli study.^54^ The most common complications were: hemorrhage (0.08‐0.18%), infection (0.06%), and injury to the penis (0.01‐0.04%).^51^

### 4.3 Deaths from EIMC

Data reporting that ≥117 newborn males died from MC in the United States each year stemmed from an assumption that the well‐known higher infant mortality in males was entirely due to MC complications.^55^ This sex difference is, however, also seen in noncircumcising countries (tabulated in reference^56^). In noncircumcising Norway, the gender difference (30%) is greater than in the United States (19%) and Israel (5%).^56^ A correlation was reported between MC rate in in the United States and sudden infant death syndrome (SIDS),^57^ but correlation does not imply causation. Death during MC can occur from uncontrolled bleeding as a result of *de novo* haemophilia in an infant with no family history. Infant MC‐related deaths are exceedingly rare, and occur mostly in nonmedical community circumcisions. Data from the US National Inpatient Sample found that during 2000‐2010, one death was recorded per 49 166 circumcisions during the first 30 days of life.^58^ The authors stressed that, “*this figure should not be interpreted as causal but correlational*” and “*may include both undercounting and overcounting*.” Deaths were most common in neonates with significant comorbidities such as cardiac disease (OR 698), pulmonary circulatory disorders (OR 170), coagulopathy (OR 160), or fluid and electrolyte disorders (OR 68) (all *P* < .001). Since the authors had access to data on deaths in uncircumcised boys, their failure to present such data was a major limitation undermining the findings.

While it is difficult to ascertain actual deaths attributable to EIMC, no deaths were found among neonatal records of 100 157 boys circumcised in US Army hospitals from 1980 to 1985.^31^ In contrast, amongst 35 929 infant males who were not circumcised, 88 (0.24%) developed a UTI, leading to meningitis in 3, renal failure in 2, and death in 2.^31^ Thus, death rate was higher in the 26.4% boys who were not circumcised. That article stated in its Discussion that no EIMC‐related deaths occurred amongst 300 000 boys born in US Army hospitals between 1970 and 1986, nor amongst 650 000 infants who had EIMC in Texas from 1971 to 1987.^31^ It further stated, “*We can find evidence for no more than two to three deaths per year that can be attributed to the procedure among the more than 1,200,000 boys that are circumcised* [in the US annually].” Other studies found no deaths.^59^ The authors noted one death from an *“at home”* procedure in records of the New York City Health Department in 1953,^60^ but there were no deaths after 500 000 EIMCs in the United States in 1982.^61^ In the mid‐1940s in England, deaths during MC of boys aged 0‐4 years were mostly from the types of general anesthetics used at the time.^62^

In Canada, where approximately half of males are circumcised, only three deaths were attributed to EIMC^63^ and three to vaccination over the period 1992‐2004.^64^ The report also documented 38 cases of anaphylaxis, 37 cases of convulsions, and 4 brain infections attributable to vaccination. Like EIMC, benefits of childhood vaccination greatly outweigh the risks. In comparison to deaths from EIMC, in Canada there were 43 deaths from penile cancer,^65^ 3708 annual deaths from prostate cancer and 443 from cervical cancer.^65^ The evidence, discussed below, shows EIMC reduces risk of each of these diseases.

### 4.4 Breastfeeding outcomes

A longitudinal study in New Zealand found that, over the course of 4 months, there was no difference in initiation of, duration of, or stopping of breastfeeding in circumcised versus uncircumcised males.^66^ Similar findings were obtained for infants from discharge to 2 weeks in a large retrospective San Diego study.^67^ No significant differences in 43 mother‐infant interactions during breastfeeding were found between neonatally circumcised and uncircumcised infants in a Missouri study.^68^ Outcomes associated with breastfeeding, such as being less prone to gastrointestinal problems and asthma, were also unaffected.^66^ Whether or not anesthesia was used for the EIMC procedures was not stated, although it would have been less likely for the New Zealand cohort of boys born in the 1970s. Those authors concluded, “*These results strongly suggest that claims about the adverse effects of neonatal circumcision on breastfeeding and child health are not sound, and have arisen as a result of unjustified extrapolation from the evidence on neonatal responses to circumcision*.”^66^

### 4.5 Meatal stenosis (MS)

MS has been said to be a common complication of circumcision.^69^ Often quoted by opponents is a prevalence of 20% reported in a small study of neonatally circumcised boys at age 5‐10 years attending a pediatric clinic in Iran for other problems, the incidental MS diagnosed being asymptomatic.^70^ MS data from a large Danish study^71^ were further evaluated by critics, revealing a MS prevalence of 0.099% in Muslim (circumcised) males and 0.12% in non‐Muslim (uncircumcised) males, of all ages combined (0‐60+ years), making the condition uncommon.^72^ A small US study that reported a figure of 7% in circumcised boys, that was not significantly higher than in uncircumcised boys,^73^ was strongly criticized by a former chair of the AAP's infant MC policy committee.^74^ In the Danish study, prevalence of other urethral stricture disease was 0.55% in Muslim and 0.82% in non‐Muslim males.^71^, ^72^ In elderly men prevalence of MS was 1.9 times higher in the uncircumcised.^71^ Each condition was higher in younger ethnic Danish men circumcised for medical problems compared with uncircumcised Danish men.^71^ Rather than being a long‐term complication of MC,^75^ onset was found to occur in the first 2 months after neonatal MC,^76^ but diagnosis is generally much later.^77^

A recent meta‐analysis of all published data from 27 studies (representing 350 MS cases amongst 1 498 536 males) found an overall summary risk estimate of 0.66% for MS in circumcised males.^78^ In uncircumcised males MS gradually increases in prevalence with age, mostly as a result of penile inflammation caused by lichen sclerosis, which is much more common in uncircumcised males.^71^, ^72^, ^78^ MS in uncircumcised males is likely underreported.^78^ Correct diagnosis can, moreover, present challenges.^79^ While more studies are warranted, the current data do not support MS being a major adverse effect of MC.

### 4.6 Glans keratinization

An argument that over time the glans of a circumcised penis becomes thickened, hardened and cornified is contradicted by histological studies comparing glans skin of circumcised and uncircumcised men.^80^, ^81^ A difference in rete ridges/pegs was, however, found in a small study,^81^ but the finding could have been confounded by age. Further research is therefore needed to clarify whether there is any effect of MC on rete ridges.

### 4.7 Psychological harm

#### 4.7.1 “False beliefs”

In a recent survey of 902 US men by MC opponents, a satisfaction score of 3.5‐3.9 out of 5 amongst 732 circumcised men was found, compared to lower scores among 170 uncircumcised men.^82^ Rather than accepting the findings at face value, the authors then asserted that circumcised men held, “*false beliefs concerning circumcision and the foreskin*,” and that, “*These findings provide tentative support for the hypothesis that the lack‐of‐harm reported by many circumcised men …. may be related to holding inaccurate beliefs concerning unaltered genitalia and the consequences of childhood genital modification*.”^82^

#### 4.7.2 Pain

It has been argued that pain associated with EIMC causes permanent, harmful, neurological changes in the brain.^83^ As support, a small study by Taddio et al found neonatally circumcised infants exhibited a stronger pain response to vaccination at 4 or 6 months than did uncircumcised infants.^84^ This finding was, however, confined to infants circumcised without anesthetic. Infants circumcised with topical local anesthesia (EMLA cream) had significantly lower pain scores at later vaccination than those circumcised without anesthetic.^84^ Taddio et al recommended there be a *“study of the vaccination pain response of infants who had received more effective circumcision pain management.”* Pain can be virtually eliminated when local anesthetic creams are applied *an hour* prior to the MC procedure.^85^

An *“after‐hours”* MRI brain scan of a single infant before and after circumcision without anesthesia was reported to reveal changes in parts of the brain associated with reasoning, perception, and emotion.^86^ Ethical approval, logistics, and compliance with procedural guidelines were not stated. The mother was strongly opposed to MC, leading critics to question her approval for this experiment and an assertion that the online report, by an MC opponent, was a fabrication.^87^ A study of 20 Jewish males in Dresden, Germany found that MC did not alter long‐term limbic‐hypothalamic‐pituitary‐adrenal axis activity, subjective stress perception, anxiety, depressiveness, physical complaints, sense of coherence, and resilience.^88^ Rather, the study found that an increase in the glucocorticoid levels indicated a healthy lifestyle and appropriate functioning, concluding that the study provided evidence that MC does not promote psychological trauma. An MC opponent commented that the study was underpowered.^89^ A larger confirmation study would help address this.

#### 4.7.3 Cognitive ability later in life

A New Zealand longitudinal study comparing boys circumcised in 1977 or left uncircumcised found no adverse effect on cognitive ability (IQ at age 8‐9 years and scholastic ability at age 13).^66^ Similarly, a Swedish study of schoolboys found no adverse psychological effect of MC.^90^ A longitudinal study in the United Kingdom, beginning in 1946, of more than 5000 individuals followed from birth to age 27, found no difference in developmental and behavioral indices between circumcised and uncircumcised males.^91^ Taken together, these consistent findings in different populations support an absence of an effect of MC on cognitive ability.

#### 4.7.4 Satisfaction and body image of boys

A study of boys aged 9‐11 in San Francisco found that circumcised boys had higher satisfaction scores, in contrast to general body image, which was no different.^92^

#### 4.7.5 Autism spectrum disorder (ASD)

Analysis of a Danish national medical records databank led to a finding that *“circumcision pain”* causes ASD and hyperkinetic disorder in a study of boys aged 0‐9 years circumcised before the age of 2 years.^93^ Critics exposed numerous flaws in the study, pointing out that the number of cases was small, statistical significance was marginal, association was stronger in Muslim boys which might suggest a need for consideration of genetic or cultural influences, association with ASD of painful conditions more prevalent in uncircumcised boys (such as cystitis) was not examined, association with ASD diagnosis was found in boys under the age of 4 years, but not in boys aged 5‐9 years, which is relevant to alternative explanations such as neuronal damage caused by analgesic usage on immature brains.^94^ General anesthesia, sometimes advocated for infant MC,^95^ is neurotoxic and associated with later cognitive impairment.^96^ It has generally been disavowed in favor of local anesthesia.^1^, ^97^ Medications for post‐EIMC analgesia—specifically, the use of acetaminophen (paracetamol), found in 1994 to be effective for management of post‐EIMC pain,^98^ led the AAP to recommend it.^99^ In support of acetaminophen use, rather than EIMC, being responsible for the association, a US study by Bauer et al found no association of EIMC with ASD prior to 1995.^100^ Unlike in older individuals, acetaminophen metabolism in immature brains generates neurotoxic by‐products. Bauer criticized the Danish ASD study for falsely suggesting that her group's findings applied to EIMC.^101^ These observations may also explain why the older boys in the Danish study (ie, boys born before the introduction of the guidelines in 1999) showed only a weak association of MC with autism, whereas the younger ones (born after 1999) showed a stronger association. Another Danish study, by Sneppen and Thorup, found an extraordinarily high prevalence of ASD of 7.2% in *uncircumcised* boys.^102^ They suggested that the figure of 1.5% reported by Frisch and Simonsen for uncircumcised Danish boys^93^ indicated confounding in the latter study. Diagnosis of ASD has been rising steady over the years but has now plateaued in males at 3.6% in the United States^103^, ^104^ and 3.7% in South Korea,^105^ whilst rate of MC has been steadily declining in each country. Other authors have also criticized the Danish autism study.^106^, ^107^

#### 4.7.6 Alexithymia

Alexithymia is an idiopathic personality trait characterized by difficulty identifying and describing an individual's own, or other peoples’ emotions. Like many personality traits, a complex interaction of genetics and environment is generally postulated to be responsible. It has been argued that early trauma, such as pain from EIMC (presumably when performed contrary to recommendations to use local anesthesia), affects the brain, leading to alexithymia.^108^ Research support for the hypothesis was provided in a study involving subjects recruited by advertisements on an anti‐MC website.^108^ Psychiatric problems appeared to be more common in men unhappy at having been circumcised.^109^ Body dysmorphic disorder has been linked to alexithymia.^110^ Consistent with bias in the small self‐selected sample, the overall rate of alexithymia was over 3 times higher than seen in the general population.^111^ There was, moreover, no association between age of MC and alexithymia. The authors later conceded that, *“Circumcision pain itself did not seem to effect* [sic!] *acquiring alexithymia,”* that their sample may be biased, and that the findings were both *“preliminary”* and needed replication.^112^

There is strong empirical support for alexithymia being a stable personality trait rather than simply a consequence of psychological distress.^113^ A large survey evaluating a comprehensive array of emotional problems in preschool^114^ and in 6‐ to 16‐year‐old^115^ children from 24 different societies found differences in severity of these between countries, irrespective of MC prevalence in each. While some, but not all,^116^ studies have shown that men exhibit higher alexithymia scores than women, the difference is seen in countries with divergent MC rates.^111^

#### 4.7.7 Psychological trauma

An unpublished study in 2000 claimed MC was associated with post‐traumatic stress disorder.^117^ This was contradicted by the survey above.^108^ We found no studies to support other MC trauma‐related claims.^118^

#### 4.7.8 Conclusion

Studies listed in Table 4 reporting negligible adverse effect of MC on physical or psychological outcomes compare favorably with those reporting an adverse effect.

**Table 4:** Quality rating^23^ of published studies that have shown negligible physical and psychological effects of MC and studies claiming a detrimental effect

Rating	Negligible adverse effect
2++	El Bcheraoui et al,^51^ Fergusson et al,^66^ Marshall et al,^68^ Morris & Krieger,^78^ Calnan et al,^91^ Bauer & Kriebel^100^
2+	Christakis et al,^53^ Wiswell & Geschke,^31^ Ben Chaim et al^54^
	Mondzelewski et al,^67^ Halata & Munger,^81^ Stenram et al^90^
	Schlossberger et al,^92^ Sneppen & Thorup,^102^ Ullman et al^88^

Rating	Detrimental effect
2+	Taddio et al^84^
2–	Frisch et al,^135^ Frisch & Simonsen,^71^ Bollinger & Van Howe^108^
4	Bollinger^55^, Adler^69^, Van Howe^73^, Tinari^86^, Boyle et al^132^

### 4.8 Sexual function and pleasure

#### 4.8.1 Sexual function

All systematic reviews of relevant research studies rated by quality found no harmful effect.^119^, ^120^, ^121^, ^122^ One systematic review included data from 19 542 uncircumcised and 20 931 circumcised men.^119^ The key finding was that MC had no adverse effect on sexual function, including erectile function, premature ejaculation, ejaculatory latency, orgasm difficulties, and pain during penetration. Evaluations by researchers in China^120^, ^121^ and Denmark,^122^ where MC is uncommon, found the same. The findings were, moreover, supported by meta‐analyses of each sexual dysfunction.^120^, ^121^ The most recent of these found pain during intercourse was 64% more common in uncircumcised males, and that that erectile dysfunction was significantly less common in circumcised men.^121^ A UK study of 6293 men and 8869 women added further support.^123^ A case‐control study in Kenya found that circumcised men reported less pain during sexual intercourse than uncircumcised control men during 2 years of follow‐up.^124^ Other aspects of sexual function did not differ between circumcised and uncircumcised men. Included in each review were 2 RCTs,^125^, ^126^ which are regarded as high‐quality evidence.^23^ Each RCT found no adverse effect on any aspect of sexual function by the 2‐year post‐MC follow‐up point. Coital injuries were significantly lower in circumcised men.^127^, ^128^, ^129^

Sexual dysfunction is common in men.^130^ There is now strong evidence that MC is not responsible, as we will present below.

#### 4.8.2 Sexual pleasure

Several studies concluded that MC diminishes sexual pleasure for men and their female sexual partners.^131^, ^132^, ^133^, ^134^, ^135^, ^136^, ^137^ Evaluation of these identified multiple flaws.^119^, ^138^, ^139^, ^140^, ^141^, ^142^, ^143^, ^144^ Other studies,^145^, ^146^, ^147^, ^148^ including RCTs,^125^, ^126^, ^127^, ^149^ found MC had no adverse effect. In fact, the RCTs found a net increase in sexual pleasure in men and their female partners. The reasons given by women for favoring MC were also esthetics, vaginal penetration, hygiene, and reduced infection risk.^149^ A systematic review of all 29 relevant publications found the same,^150^ as did a smaller systematic review.^151^

A list of “*16 functions of the foreskin*”^152^ (Table 3) compiled by opponents, and widely circulated on the Internet will now be evaluated in relation to data, when available, there being no evidence to assess the veracity of some of the claims.

It has been argued that the foreskin contains “*10 000*” or *“20 000*” nerve endings essential for sexual pleasure. The *“10 000*” figure (specifically fine‐touch nerve endings; Meissner's corpuscles) stemmed from a calculation by Prof. Ken McGrath, which he subsequently retracted as being, “*an order of magnitude too high*.”^153^

Fingertips have the highest concentration of Meissner's corpuscles of any human glabrous skin, and the foreskin the lowest.^154^ Meissner's corpuscles in the foreskin are most abundant up to age 10‐14 years, then decline,^155^ which was stated, by Cox et al, to contradict the sexual pleasure claim.^143^ Cox et al provided data explaining that other types of nerve endings specific to the glans, but absent from the foreskin, are responsible for sexual pleasure.^143^

The figure of *“20 000*” nerve endings appeared in a 1997 magazine article^156^ by Paul Fleiss.^157^ It cited as support a 1932 paper^158^ that did not state there are 20 000 nerve endings in the foreskin. Instead, the *“20 000*” figure stemmed from a count of 212 nerve endings in 1 cm^2^ of an undisclosed part of a single foreskin from an individual of unknown age.^158^ Amongst these were 2 fine‐touch receptors, but no genital corpuscles that have been invoked as the nerve endings responsible for erogenous sensations.^143^ To arrive at “*20 000*,” 212 would need to be multiplied by 94.3. The 94.3 cm^2^ value for both inner and outer surfaces combined is near the top of the range of 7‐99.8 cm^2^ (av. 38.5 cm^2^) reported more recently for total foreskin surface area.^159^

It has also been argued that the foreskin has a surface area of *“15 square inches.”* 21 This value is at the upper (∼0.1%) limit of the range found for the combined inner and outer foreskin area of 965 Ugandan men (aged 15‐49 years) of 7‐99.8 cm^2^ (mean 38.5 cm^2^),^159^ that is, 1.1‐15.5 square inches. The only other study, involving 8 cadavers (of unstated age, race, etc), reported a combined outer and inner foreskin area of 18.1‐67.5 cm^2^ (2.8‐10.5 square inches, mean 7.2 square inches [46.7 cm^2^]).^160^ Those measurements showed that foreskin size is highly variable, very much more so than penis length.^161^ Darwin noted, *“An organ, when rendered useless, may well be variable, for its variations cannot be checked by natural selection.”* 162

We could find no evidence to support the claim of pheromones being present in the foreskin.^163^

It has been postulated that, “*In heterosexual intercourse, the non‐abrasive gliding of the* [uncircumcised] *penis in and out of itself within the vagina facilitates smooth and pleasurable intercourse for both partners*,” meaning easier penetration, nerve stimulation and prevention of loss of vaginal lubricant.^164^ No gliding would, however, occur for men with short foreskins. We could find no studies investigating this proposed phenomenon in men or their sexual partners. The purported lubrication provided by “*gliding*” should reduce pain during intercourse (dyspareunia). However, most studies reported either no difference or less pain in *circumcised* men,^119^, ^120^, ^121^, ^122^, ^124^, ^125^, ^126^, ^127^, ^165^ and their female sexual partners^149^, ^150^ (Table 5). Contrary claims appeared to be based on speculation, anecdotes, or low‐quality studies.^166^, ^167^

**Table 5:** Pain during sexual intercourse for circumcised vs uncircumcised men, and for women with circumcised vs uncircumcised partners

Reference	Type of study	n	More (+), less (–), no difference (0)
**Men**			
Kigozi et al, 2008^125^	RCT	1500	0
Krieger et al, 2008^126^	RCT	1995	0
Morris & Krieger, 2013^119^	Systematic review	8288 vs 6894 (6 studies)	0 (all 6 studies)
Tian et al, 2013^120^	Systematic review & meta‐analysis	7349 vs 6407 (5 studies)	0 (4 studies); – (1 study)
Shabanzadeh et al, 2016^122^	Systematic review	8 studies	0 (7 studies); – (1 study)
Brito et al, 2017^127^	Cohort study	500	– (*P* < .001; fewer coital injuries)
Galukande et al, 2017^165^	Cohort study	304	– (42%); 0 (58%)
Nordstrom et al, 2017^124^	Case‐control	>3000	– (*P* < .001).
Yang et al, 2017^121^	Systematic review & meta‐analysis	6736 vs 4201 (6 studies)	0 (3 studies); – (3 studies)
**Women**			
Kigozi et al, 2009^149^ (*P *= NS)	RCT	455	0 (99.8%); + (0.2%)
Morris et al, 2019^150^	Systematic review		0 (3 studies); – (2 studies); + (1 study)

Further information addressing the “16 functions” is available.^168^

#### 4.8.3 Data from high‐quality studies

Two high‐quality studies, a RCT in Kenya^126^ and a cohort study in the Caribbean,^127^ found that most sexually experienced men reported improved sexual pleasure and function after circumcision. A meticulously conducted systematic review of all studies found that, overall, MC had no adverse effect on penile sensitivity, sexual arousal, sexual sensation, or pleasure.^119^ Criticisms of that study^137^ were shown to lack merit.^142^ The findings were consistent with a systematic review of histological correlates of sexual sensation showing that the sensory receptors responsible for sexual pleasure (genital corpuscles) reside in the glans, not the foreskin, meaning loss of the foreskin by MC should not diminish sexual pleasure.^143^ By exposing the glans, as often occurs in an uncircumcised man during erection, MC was proposed to increase sexual pleasure.^143^ The foreskin, just as other skin on the body, contains sensory receptors that respond to touch, temperature and pain. Since the density of Meissner's corpuscles in the foreskin diminishes at puberty when male sexual activity is increasing, these touch receptors are unlikely to be involved in sexual sensation.^143^ Moreover, free nerve endings (that respond to touch) showed no correlation with sexual response. Sensitivity of the glans to touch decreased with sexual arousal, so further diminishing a role for touch receptors in sexual sensation.^169^ Sensitivity of the penis to vibration, which is able to elicit arousal and ejaculation, is not related to MC status.^143^

#### 4.8.4 “Foreskin restoration”

This undertaking involves stretching the skin on the shaft of the circumcised penis using weights. Various psychological disorders^170^, ^171^ were found to be more prevalent in circumcised men preoccupied with their absent foreskin.^109^ Such men were more likely to undertake *“foreskin restoration,”* which was found to occasionally require subsequent “*re‐circumcision*”^172^, ^173^ or medical attention for resulting genital mutilation.^172^, ^174^

#### 4.8.5 Conclusion

As summarized in Table 6, high‐quality research shows that MC has no adverse effect on sexual function, sensitivity, or pleasure. This finding contradicts arguments based on low‐quality evidence.

**Table 6:** Conventional quality rating^23^ of published studies that have shown no adverse effect of MC on sexual function and pleasure and of studies finding a detrimental effect

Rating	Studies showing no adverse effect
1+	Tian et al,^120^ Nordstrom et al,^124^ Kigozi et al,^125^, ^149^ Krieger et al^126^
1–	Morris & Krieger,^119^ Cox et al,^143^ Yang et al,^121^ Shabanzadeh et al,^122^ Payne et al^169^
2++	Homfray et al,^123^ Brito et al,^127^ Galukande et al,^165^ Bossio et al^144^
2+	Cortés‐González et al,^146^, ^147^ Zulu et al^148^

Rating	Studies showing a detrimental effect
2–	O'Hara & O'Hara,^131^ Boyle & Bensley,^132^ Kim & Pang,^133^ Sorrells et al^134^ Frisch et al,^135^ Bronsalaer et al^136^

### 4.9 HIV infection

#### 4.9.1 In heterosexual men

Evidence showing that MC provides protection against heterosexually acquired HIV infection in men has been disputed.^30^, ^175^, ^176^, ^177^, ^178^, ^179^, ^180^, ^181^, ^182^, ^183^, ^184^, ^185^, ^186^, ^187^, ^188^, ^189^, ^190^, ^191^, ^192^ Early evidence of protection^193^ was confirmed by three RCTs in sub‐Saharan Africa,^194^, ^195^, ^196^ a review,^197^ and a Cochrane committee meta‐analysis that showed high consistency of the trial results,^198^ leading to endorsement of MC by the World Health Organization (WHO) and UNAIDS as an additional important intervention to help reduce HIV prevalence in epidemic settings.^199^, ^200^ Roll‐out of VMMC programs has resulted in 18.6 million MC procedures in high‐priority countries.^201^ VMMC has been very effective in lowering HIV infections in epidemic settings in sub‐Saharan Africa.^202^, ^203^, ^204^, ^205^, ^206^ In a recent Kenyan study the reduction was 50%.^206^ Criticisms of the RCT findings by MC opponents^176^, ^177^, ^178^, ^179^, ^188^, ^189^, ^190^, ^191^, ^192^ were shown by scientists and public health authorities to contain fundamental flaws.^56^, ^207^, ^208^, ^209^, ^210^, ^211^, ^212^, ^213^, ^214^, ^215^, ^216^, ^217^, ^218^, ^219^, ^220^, ^221^, ^222^, ^223^ The suggestion that, once circumcised, men would forego condom use was contradicted by a recent meta‐analysis that found no difference in condom use for up to 2 years post‐MC.^224^

Recent meta‐analyses have shown HIV protective effects of MC in circumcised men of 70% (95% CI 0.24‐0.38; *P *< .00001)^225^ and 72% (95% CI 1.7‐7.1).^226^

Compelling biological reasons explain the vulnerability of the foreskin to HIV infection.^80^, ^227^, ^228^, ^229^, ^230^, ^231^ Infectivity is exacerbated in inflammatory states and ulceration from sexually transmitted infections (STIs),^232^, ^233^, ^234^, ^235^, ^236^ coital injuries (more common in uncircumcised men),^127^, ^128^, ^129^ and foreskin size.^159^ Langerin, produced by the mucosal epithelium of the foreskin, is protective at low viral loads,^237^ but becomes overwhelmed at high HIV loads.^237^, ^238^

Those who had denied the evidence, but have now accepted that MC is effective in HIV prevention in sub‐Saharan Africa, continue to dispute its effectiveness in developed countries, despite US data confirming that MC protects men in the United States against HIV during heterosexual intercourse,^239^, ^240^ supported by the US CDC.^3^, ^241^ In countries with comparable sexual behavior indices, condom use and access to HIV testing and treatment, those with low MC prevalence (the Netherlands and France), had annual rates of new heterosexually acquired HIV diagnoses that were 6 times higher in men and 10 times higher in women than in Israel, where MC prevalence is high.^242^

#### 4.9.2 HIV infection in women

Based on data from two studies,^243^, ^244^ it was argued that MC increases women's HIV infection risk. In the Rwanda study, women with higher HIV‐positivity were from higher socioeconomic groups^243^ in which MC is more common, as is promiscuity. Cross‐infection from unhygienic traditional MC may also have contributed.^245^ The Uganda study found that 17 women in the intervention group (18%) and 8 (12%) in the control group acquired HIV during follow‐up (*P *= .04). The marginally higher HIV infection in the female partners of men who had been circumcised was limited to women whose male partner disobeyed medical advice and resumed sexual intercourse prior to the end of the 6‐week post‐MC wound‐healing period.^244^ Inadequate recruitment, and thus power, resulted in the trial being stopped at interim analysis.^244^ Enrolment of the necessary 10 000 serodiscordant couples was deemed “*logistically unfeasible*.”^246^

Meta‐analyses have found a 20%^246^ and 32%^225^ nonsignificantly lower HIV risk in women with circumcised male partners. HIV prevalence was 78% lower (*P* = .035) in South African women who only had circumcised male partners.^247^ Recent systematic reviews have documented all MC and HIV studies in women.^248^, ^249^

#### 4.9.3 HIV infection in men who have sex with men (MSM)

A Cochrane meta‐analysis concluded that, “*Current evidence suggests that male circumcision may be protective among MSM who practice insertive anal sex, but the role of male circumcision overall in the prevention of HIV* […] *among MSM remains to be determined*.”^250^ The meta‐analysis found a 73% decrease in HIV infection risk in studies of MSM reporting an *insertive* role during anal intercourse, but no significant difference in studies of men reporting a receptive role.^250^ A more recent meta‐analysis, involving 62 observational studies and 119 248 MSM, found MC was associated with a significant, overall 23% reduced risk of HIV infection among MSM (OR 0.77; 95% CI 0.67‐0.89).^251^ the implications of which were further discussed in an accompanying editorial.^252^ Each meta‐analysis referred to the highly significant 89% risk reduction conferred by MC to insertive MSM in Sydney, Australia,^253^ and called for more studies of MSM who adopt the insertive role during anal intercourse, as well as more studies of bisexual men because of the risk of STI transmission they pose to women.

#### 4.9.4 Intercountry comparisons

Arguments that HIV rate is higher in the United States than Europe despite higher MC rate in the United States, have failed to acknowledge that the major route of HIV infection in the United States is receptive anal intercourse amongst MSM, for which MC affords no protection.^56^

### 4.10 Other sexually transmitted infections

#### 4.10.1 Overview

An extensive article disputed the ability of MC to protect against STIs other than HIV.^254^ Detailed evaluation of that article revealed serious flaws in statistical analyses, as well as obfuscation and misrepresentation of data.^255^ The author, Robert Van Howe, has a history of analyses of MC and other STIs^254^, ^256^, ^257^, ^258^ that have been shown to contain serious analytical and evidential flaws.^255^, ^259^, ^260^

The following summarizes the high‐quality evidence addressing the role of MC in protecting against various specific STIs.

#### 4.10.2 Oncogenic human papillomavirus (HPV) genotypes

A recent meta‐analysis of 30 studies found MC was strongly associated with reduced odds of genital HPV prevalence (OR 0.68; 95% CI 0.56‐0.82).^261^ That meta‐analysis treated all study types equally. Risk reduction was 53‐65% in 2 earlier meta‐analyses and 40% in 6 RCTs.^262^, ^263^, ^264^, ^265^, ^266^, ^267^ (See also recent risk‐benefit analyses^268^, ^269^.) A large multinational study found penile HPV in 19.6% of uncircumcised versus 5.5% of circumcised men.^270^ After adjustment for age at first intercourse, lifetime number of sexual partners, and other potential confounders, circumcised men were 63% less likely to be infected with HPV.^270^ A large UK survey found high‐risk HPV types were 86% less prevalent in uncircumcised men.^271^ A RCT published in 2012 found that the incidence of flat penile lesions (mostly caused by high‐risk HPV types) was 98% lower among circumcised men.^262^ Thus, high‐quality studies and analyses confirm the protective effect of MC against high‐risk HPV types.

MC also protects against low‐risk (nononcogenic) HPV types responsible for genital warts.^272^ These HPV types infect the shaft and genital area generally, whereas high‐risk types mostly infect the foreskin and underlying glans.^272^ A RCT found that circumcised men had a shorter duration of HPV infection of the glans/coronal sulcus,^273^ but duration of infection did not vary by circumcision status in the penile shaft, scrotum, or all genital sites combined. Thus, clearance is greatest in precisely the area of the penis exposed by MC. A US study found that MC was associated with a statistically significant increased likelihood of clearance of any HPV infection (HR 2.7; 95% CI 1.3‐5.7) and of clearance of oncogenic HPV infection (HR 3.2; 95% CI 1.4 ‐7.4]), but not with an increased clearance of nononcogenic HPV infection.^274^ The meta‐analysis cited above conceded that, *“sampling sites also played an important role in the final results”* and that, *“selection bias in our meta‐analysis”* (ie, not taking into account penile sites used for sampling) affected the conclusions.^261^ Use of a single combined sample for the penis and scrotum was the likely explanation for a negative result in one study.^275^ Foreskin HPV infection is significantly higher in men with phimosis.^276^

In summary, MC reduces penile infection by, and increases clearance of, high‐risk HPV genotypes.

#### 4.10.3 Genital herpes simplex virus‐2 (HSV‐2)

Data from 3 RCTs in sub‐Saharan Africa found significant decreases of 45%, 30%, and 28% in HSV‐2 infection in men after MC.^277^, ^278^, ^279^, ^280^ A 2006 meta‐analysis, that predated publication of the RCTs, found HSV‐2 was 15% (OR 0.74‐0.98) lower in circumcised men, after adjustment for confounding factors.^281^

#### 4.10.4 Protection of men against other STIs

As documented in a critical review,^255^ RCTs and other studies have found MC affords protection against *Trichomonas vaginalis* (50%),^282^ *Mycoplasma genitalium* (40%),^283^ *Treponema pallidum* (syphilis) (33‐50%),^281^, ^284^, ^285^ chancroid (50%),^281^ and genital ulcer disease (50%).^286^, ^287^ Genital ulcers in uncircumcised men contain a higher prevalence of anaerobic bacteria. RCT data showed that MC reduces total bacterial load and microbiota biodiversity.^49^ A RCT found no syphilis infections in the 24 months after MC compared with 9.6% in men who remained uncircumcised (*P *= .09).^232^ Although RCT data by Tobian et al failed to find a reduction in syphilis, this might have reflected lack of statistical power due to the small number of syphilis infections identified on follow‐up testing.^288^ Tobian, in an editorial covering another large study that found 42% lower syphilis in circumcised men,^284^ acknowledged that MC *does* reduce syphilis risk.^289^ Arguments disputing the use of MC for syphilis risk reduction^290^ have been criticized as flawed.^219^ Data show that MC does not protect men against sexually transmitted urethritis.^260^

#### 4.10.5 Protection against STIs in women

Findings on the impact of MC on STIs in women are mixed. At the very least, it should be obvious that any measure that reduces risk to the male partner of being infected should reduce STI prevalence in women. Below we summarize available data.

In women, high‐risk HPV infection may cause cervical dysplasia that can progress to cervical cancer. High‐risk HPV also contributes to other genital cancers and to oropharyngeal cancers. Over her lifetime, a woman may have sexual partners of either MC status, potentially confounding associations between male partner MC status and a woman's HPV risk. This issue was addressed in a large multinational study, in which confounding was minimized by restricting the analysis to 1420 men whose female partner reported having had only a single sexual partner.^270^ The men were rated for their “sexual‐behavior risk index.” Men who were high‐risk had had ≥6 sexual partners and first intercourse prior to 17 years of age. Men who were low‐risk had had ≤5 sexual partners and first sexual intercourse at >17 years of age. The remaining men were classified as having an intermediate risk. Monogamous women whose male partner had either a high or an intermediate sexual‐behavior risk index were much less likely to have had a cervical cancer diagnosis if the male partner was circumcised (OR 0.18 [95% CI 0.04‐0.89] and 0.50 [95% CI 0.27‐0.94], respectively). A MC RCT found that after 2 years the incidence of high‐risk HPV infection in women was lower in those women whose male partners were in the circumcised group than in women whose male partners were in the control group (20.7 infections vs 26.9 infections per 100 person‐years; incidence rate ratio = 0.77‐0.63‐0.93; *P *= .008).^291^

An argument that effective HPV vaccines render MC for protection irrelevant fails to appreciate that current HPV vaccines are prophylactic not therapeutic, are primarily administered to girls (and more recently boys) in early high school, are not directed at all of the > 14 mucosotropic HPV genotypes, and that overall vaccine uptake in females aged 10‐20 is only 33.6% in high‐income countries, and 2.7% in low‐ and middle‐income countries.^292^ In Australia, one of the first countries to introduce a national HPV vaccination program, the 3‐dose coverage for girls turning 15 years of age in 2016 was 78.6% and in boys was 72.9%.^293^ In theory, the recent introduction (in Australia) of a nonavalent HPV vaccine to replace the quadrivalent HPV vaccine could, only if 100% effective, increase protection from 70% to as much as 93% if vaccine coverage in school children is universal. A recent systematic review of real‐world experience with HPV vaccination^294^ revealed its suboptimal effectiveness (see figure 3C of that publication). In Australia, one of the earliest countries to vaccinate girls (in 2007), there was an 86% (not 100%) decrease in the four *vaccine genotypes* (HPVs 6, 11, 16, and 18).^294^

As with other public health interventions, a package of multiple preventive measures is likely to have a greater impact than vaccination alone. HPV vaccination against a subset of HPV types in early adolescence can help mitigate cervical cancer risk, but uptake is not widespread in all settings and durability of effectiveness remains to be seen. The emerging switch from pap smears to primary screening for HPV in high‐income countries, by a PCR‐based test,^295^, ^296^ will improve risk detection, but is not practicable in resource‐constrained settings.

Genital herpes infection risk in a Pittsburgh study was twice as high in women who had ever had intercourse with an uncircumcised man (OR 2.2; 95% CI 1.4‐3.6; n = 1207).^297^ Similarly, a RCT found 2‐fold higher HSV‐2 infection over 12 months in 783 wives of uncircumcised men.^298^ Secondary data from another RCT found HSV‐2 was the primary pathogen in 96% of the 67% of genital ulcers in the female partners in whom an etiological agent had been identified.^299^ Most participants had been infected with HSV‐2 prior to commencement of the trial and HSV‐2 detected in these women represented mostly reactivation of preexisting infection.

*Chlamydia trachomatis* seropositivity in a large, multinational study was 5.6‐fold higher in women with an uncircumcised male partner.^300^ The finding also applied to women who had only had one sexual partner. Prevalence of *C. pneumoniae*, which is not transmitted sexually, did not differ. The authors speculated that infected cervicovaginal secretions may be trapped under the foreskin for longer in uncircumcised men, increasing risk of penile urethral infection and transmission to the vagina during sexual intercourse.^300^ A prospective study involving populations from Uganda, Zimbabwe, and Thailand, however, found no difference in chlamydial, gonococcal, or trichomonal infections in women as a function of MC status.^301^

For other STIs, a RCT found that genital ulcer disease risk was 22% lower in women with circumcised male partners, bacterial vaginosis was 40% lower, severe bacterial vaginosis was 61% lower, and *Trichomonas vaginalis* was 48% lower, but there was no difference in dysuria or vaginal discharge.^298^ A large prospective cohort study of 2946 HIV‐negative couples found syphilis was 75% lower in the female partners of circumcised men.^284^ A prospective study in Kenya by the same authors found that those with circumcised male partners had a 58% lower risk of incident *Trichomonas vaginalis* compared to women with uncircumcised partner.^302^

A recent systematic review of MC and STIs in women identified 9 RCTs and 48 observational studies of populations globally.^249^ Overall, MC reduced acquisition of STIs and cervical cancer in women, being strongest for HSV‐2, chlamydia, and syphilis. The authors found medium consistency evidence for protection against any HPV type and low‐risk HPV types, intermediate consistency for any STI, candidiasis, dysuria, genital warts, gonorrhoea, high‐risk HPV viral load, and *Mycoplasma genitalium*, with discrepant values for bacterial vaginosis, HIV, high‐risk HPV, nonspecific genital ulcers, trichomonas, and vaginal discharge that rendered the latter low consistency. More information was presented in an editorial.^303^ Another recent systematic review identified 82 studies of MC and STI in women, leading to similar conclusions.^248^ Clearly, reduced population prevalence of STIs in men will translate into lower risk of STI exposure in women.

#### 4.10.6 Protection against other STIs in MSM

A study in 2012 found that MC provided 57% protection against the major oncogenic HPV type, HPV16, in Australian MSM who practiced predominantly insertive anal intercourse.^304^ Not surprisingly, no protection was observed for men who predominantly assumed the receptive role during anal intercourse. A 2011 Cochrane meta‐analysis examined syphilis, HSV‐1, and HSV‐2 in MSM and found no overall significant association with MC status.^250^ A more recent meta‐analysis found MC was associated with reduced odds of HSV infection among MSM overall (OR 0.84, 95% CI 0.75‐0.95), and of penile HPV infection among HIV‐infected MSM (OR 0.71, 95% CI 0.51‐0.99).^251^ The Australian group found that MC protected against *incident* syphilis (HR 0.35; 95% CI 0.15‐0.85), particularly in the one‐third of MSM in the study who engaged predominantly in insertive anal intercourse (HR 0.10; 95% CI 0.01‐0.81).^305^ An explanation for association with incident but not prevalent syphilis in that study was that MSM who initiated sexual activity during the late 1980s and 1990s when syphilis prevalence was low would have been at very low risk of acquiring syphilis irrespective of their MC status, but only since 2001 has syphilis re‐emerged in Australian MSM.^305^

#### 4.10.7 Conclusion

As summarized in Table 7, high‐quality data show that MC protects against risk of HIV and various other STIs.

**Table 7:** Quality rating^23^ of studies that have found MC protects against HIV and several other STIs and studies showing no protective effect

HIV	
Rating	Studies supporting a protective effect of MC
1++	Auvert et al,^194^ Bailey et al,^195^ Gray et al,^196^ Siegfried et al^198^
1+	Weiss et al,^197^ Morris et al,^221^ Lei et al,^225^ Sharma et al,^226^ Freeman et al,^233^ Weiss et al,^246^ Wiysonge et al,^250^ Yuan et al^251^
2++	Morris et al,^220^ Gray et al,^232^ Boily et al^223^, ^234^
2+	Warner et al,^239^ Sansom et al,^240^ Chemtob et al,^242^ Templeton et al^253^
2–	Wawer et al^244^

Rating	Studies disputing the protective effect of MC
2–	Van Howe^175^, ^191^, ^192^

Other STIs	
Rating	Studies supporting a protective effect of MC
1+	Waskett et al,^260^ Zhu et al,^261^ Mehta et al,^283^, ^287^ Weiss et al,^291^ Gray et al,^298^ Brankin et al,^299^ Yuan et al^251^
2++	Morris et al,^269^ Castellsagué et al,^270^, ^300^ Homfray et al,^271^ Backes et al,^262^ Hernandez et al,^273^ Lu et al,^274^ Albero et al,^275^ Tobian et al,^277^, ^278^ Sobngwi‐Tambekou et al,^279^, ^282^ Mehta et al,^280^ Pintye et al,^284^, ^302^ Otieno‐Nyunya et al,^285^ Nasio et al,^286^ Poynten et al,^304^ Templeton et al^305^
2+	Turner et al^301^

Rating	Studies disputing the protective effect of MC
2–	Van Howe^254^, ^256^, ^257^, ^258^

### 4.11 Condoms for protection against STIs

It has been argued that condoms afford complete protection against HIV and other STIs, so obviating the need for MC.^69^, ^188^, ^189^ Current data show, however, that condoms provide protection against HIV infection that ranges from 80%^306^ to 71‐77%.^307^ This protection only applies if condoms are used consistently and correctly.^306^, ^308^ Condoms may break. A Cochrane systematic review and meta‐analysis of RCTs of condom use (two in the United States, one in England, and four in Africa) found, *“little clinical evidence of effectiveness”* and no, *“favorable results”* for HIV prevention.^309^ That study did, however, find that condoms were 42% effective in prevention of syphilis infection.^309^

Unlike condoms, MC is a one‐off procedure that does not require future compliance each time a man has sexual intercourse. In this respect MC can be compared with vaccination. However, besides the hepatitis B vaccine, the only vaccines currently in widespread use (in early high school females and increasingly in males) for STI prevention are those that protect against certain HPV genotypes. MC and condom use each provide a reasonable degree of protection against STIs. When both are in place protection is higher.^56^

### 4.12 Delay of MC until males become sexually active

It has been argued that MC be delayed to allow the male to decide if he wishes to reduce his risk by choosing to get circumcised when he is old enough to be sexually active.^310^, ^311^ Substantial problems with this argument have been enunciated^312^ (Table 8). First, MC has other benefits besides STI prevention and these benefits start early in life (see UTIs section above and inflammatory skin conditions and physical problems sections below). The benefit‐to‐risk ratio from EIMC is high and has increased over the years as more evidence has accumulated^268^, ^269^, ^312^, ^313^, ^314^, ^315^, ^316^ (Table 9). Second, EIMC is simpler, quicker, less expensive, with lower risk of complications,^51^ healing is faster, and the scar can be almost invisible.^312^ Third, there are substantial barriers to later circumcision.^312^ These barriers include the decision process, peer pressure, affordability, slower healing, pain during nocturnal erections, the need to abstain from sexual activity for ∼6 weeks, and a visible scar afterwards. The sexual abstinence period is often cited by men as a significant barrier, so favoring EIMC as the preferred time.^317^ Because these barriers deter many men from getting circumcised a much higher uptake of MC can be achieved for EIMC.^318^

**Table 8:** Reasons why early infancy is the preferred time for MC

EIMC	MC of older boys and men
Simple Quick (takes several minutes) Cost is lower Low risk (adverse events 0.4%) Bleeding (uncommon) is minimal and easily stopped Sutures not needed Convenient for patient (sleeps mostly) Local anesthesia for < 2 months Healing is fast (< 2 weeks) Cosmetic outcome usually good No long‐term memory of procedure Does not disrupt (breast) feeding or other day‐today activities	More complex Half an hour or more to perform Much more expensive (often unaffordable) Moderate risk (adverse events 4‐8%) Bleeding more common, requiring cautery or other interventions Sutures or tissue glue needed Inconvenient (time off school or work) General anesthesia for > 2 months to age 9 years. Local anesthesia for men, although general anesthesia sometimes preferred by surgeon Healing takes 6 weeks or more If stitches used stitch marks may be seen Fear of undergoing an operation Abstinence from sexual intercourse for the 6‐week healing period

**Table 9:** Risk‐benefit analyses of EIMC and medical conditions over the lifetime

Benefit‐to‐risk ratio	Uncircumcised males affected	Publication
> 100:1	∼1 in 3	Morris et al 2006 *ANZ J Public Health* 314
> 100:1	∼1 in 3	Morris 2007 *BioEssays* 313
“Very favorable”	∼1 in 2	Morris et al 2012 *Open J Prevent Med* 315
“Strongly favors”	∼1 in 2	Morris et al 2012 *BMC Pediatr* 312
> 100:1	∼1 in 2	Morris et al 2014 *Mayo Clin Proc* 316
∼ 100:1	∼2 in 3	Morris et al 2016 *Can J Urol* 268
∼ 200:1	∼2 in 3	Morris et al 2017 *World J Clin Pediatr* 269

An argument that infant MC should be banned, discouraged, or at least delayed until the boy is old enough to decide for himself^18^, ^19^, ^20^, ^319^, ^320^ was refuted by authorities in ethics, who have presented sound reasons why such reasoning is flawed.^321^, ^322^, ^323^, ^324^, ^325^, ^326^, ^327^. It was argued that being circumcised boosts autonomy more than constraining it.^328^ The AAP recommended that prior to or early in a pregnancy the medical practitioner should provide parents with unbiased education about risks and benefits of EIMC so they have adequate opportunity to choose what is in their child's best interests should they have a boy.^1^ Furthermore, MC later in life is not only associated with a 10‐ to 20‐fold higher risk of adverse events,^51^ but, as explained above, having MC performed later poses significant barriers to adolescent boys and men that usually mean MC will not happen, except for a medical reason.^312^

### 4.13 Penile inflammatory conditions and treatment

There has been a trend away from MC and toward use of steroid creams for treatment of phimosis and penile inflammation.^329^ This approach is not ideal.^330^, ^331^ Commitment is needed for regular application, there is a risk of side effects from long‐term use of steroids, and effectiveness of 2 (range 1‐23) months’ treatment was only 35% during 4 (range 1.5‐60) months’ follow‐up in a recent meta‐analysis of the very serious foreskin‐related inflammatory condition, lichen sclerosus.^331^ In contrast, MC is close to 100% effective.^332^ Preputioplasty can also be used, but is less effective as a cure than MC, and serves to accommodate the wishes of those patients who want to preserve their foreskin.^333^

Phimosis, balanitis, and candidiasis can occur alone, or can co‐occur. A meta‐analysis found 68% lower balanitis rates in circumcised males.^334^ Penile candidiasis was reported in 7.7% of uncircumcised men versus 4.9% of circumcised men in a large Australian survey.^335^ In boys aged 8 months to 18 years (mean 6.4 years), the prevalence of fungal infection was 44% in uncircumcised boys versus 18% in circumcised boys.^336^ The fungal species were, in order of decreasing prevalence: *Malassezia globosa*, *M. furfur*, *M. slooffiae*, *C. albicans*, *C. tropicalis*, and *C. parapsilosis*. Each was present in uncircumcised infants, but none were present in circumcised infants. A gradual accumulation with age occurred, by age 18 years reaching 62.5% in uncircumcised boys versus 37.5% in circumcised boys. Recently, a strong direct link has been found between *C. albicans* antibodies and schizophrenia in men, independent of potential confounders.^337^

### 4.14 Penile cancer

Despite strong evidence for MC, especially EIMC, conferring protection against penile cancer, contrary arguments have been presented.^26^, ^338^, ^339^ Those arguments have been criticized.^340^, ^341^, ^342^ For example, it has been stated that because penile cancer diagnosis in men is 1 in 100 000 the disease is very rare. This figure is, however, an approximation of the *annual incidence*. The more relevant figure is *lifetime risk*, which is approximately 1 in 1000 for an uncircumcised man.^343^ This would make penile cancer uncommon, but not rare. Its prevalence in *circumcised* men, of 1 in 50 000 to 1 in 12 000 000,^344^, ^345^ might be considered rare. A California study found that uncircumcised men had a 22‐fold higher risk.^346^ The reason why uncircumcised men are at elevated risk stems from foreskin‐related conditions, most prominently phimosis, which was shown in a meta‐analysis to increase the risk 12‐fold.^334^ EIMC eliminates lifetime risk of phimosis. Meta‐analyses found that balanitis increases penile cancer risk 3.8‐fold and smegma (a whitish film that accumulates under the foreskin of men and that comprises dead and decomposing exfoliated skin cells, bacteria, and other microorganisms) increases penile cancer risk 3.0‐fold.^334^

Penile inflammatory conditions are much more common in uncircumcised men.^330^ A meta‐analysis found 47% of penile cancers are positive for high‐risk HPV genotypes.^347^ Since HPV genotypes prevented by current HPV vaccines constitute approximately 70% of population prevalence of all high‐risk HPV genotypes, one might predict that HPV vaccination would offer the potential to reduce penile cancer by up to 47 × 0.7 = 33%. This level of risk reduction is similar to that conferred by MC in a meta‐analysis^261^ and RCTs.^262^, ^263^, ^264^, ^265^, ^266^, ^267^ An early concern was that, over time, nonvaccine HPV genotypes might replace vaccine genotypes.^348^ There is now evidence for this. Eight years after introduction of the HPV vaccination program for girls in Australia, prevalence of HPV 16 and 18 decreased in heterosexual men from 13% to 3% (*P* < .0001).^349^ But there was no decrease in HPV genotypes overall, and, *“prevalence of nonvaccine‐targeted genotypes”* increased from 16% to 22% (*P* < .0001).^349^ A combination of public health measures is normally advocated for disease prevention.

### 4.15 Prostate cancer

Prostate cancer affects ≥10% of men over the lifetime. A 2015 meta‐analysis found that, after reducing heterogeneity by removing outlier studies, prostate cancer risk was significantly lower in circumcised men, especially in the post‐PSA testing era (*P *= .01).^350^ In men of African descent, large US^351^ and Canadian^352^ studies showed risk reductions of up to 36% (95% CI 8‐61) and 60% (95% CI 0.19‐0.86), respectively. MC prevalence worldwide is inversely correlated with prostate cancer incidence.^353^ Countries with high MC prevalence have lower prostate cancer‐related mortality, corrected for potential confounding factors.^354^ The risk reduction associated with MC is on a par with other commonly recognized factors associated with decreased prostate cancer risk.^355^, ^356^

### 4.16 Ethical and legal issues

Legal, human rights and other arguments (presented below) have been invoked in opposing EIMC.^26^, ^69^, ^320^, ^357^, ^358^, ^359^, ^360^, ^361^, ^362^ Evaluation of those arguments have revealed flaws.^342^, ^363^, ^364^, ^365^, ^366^, ^367^, ^368^, ^369^, ^370^, ^371^, ^372^, ^373^, ^374^ Arguments criticizing the AAP's policy statement on ethical and legal grounds^1^, ^2^, ^26^, ^311^, ^320^, ^357^, ^375^, ^376^ were followed by evaluations undermining the arguments used.^363^, ^364^, ^367^, ^374^, ^377^, ^378^, ^379^ Articles critical of the CDC's draft recommendations in 2014^69,329,358^ have also been shown to contain serious flaws.^365^, ^366^, ^368^ In its 2018 final statement,^3^ the CDC provided responses to public comments by MC opponents to the CDC's draft recommendations, repudiating most.^380^

Scholarly assessments concluded that MC of minors is ethical.^321^, ^323^, ^324^, ^326^, ^327^, ^371^, ^381^ Given the wide‐ranging protection against multiple medical conditions and infections in infancy and childhood, including STIs in adolescents who become sexually active, it was argued that it would be unethical to leave boys uncircumcised.^323^, ^371^ It was argued that Article 24(3) of the United Nations (UN) on the Rights of the Child might be interpreted as mandating EIMC, since not circumcising boys has been deemed as prejudicial to their health.^323^

The statement “*First do no harm*” (often incorrectly attributed to the Hippocratic Oath) has been used to argue against MC. That statement is derived from the four pillars of medical ethics. The argument presupposes that MC is harmful, meaning that it is based on a false premise. It ignores the principal of beneficence—acting in the patient's best interest. The statement in the Hippocratic Oath, “*I will prevent disease whenever I can, for prevention is preferable to cure*”^382^, ^383^ is pertinent. Recent evaluation of the legal and ethical issues was provided by professors of law, bioethics, urology, medicine, and medical sciences in the *International Journal of Children's Rights* 366 and the *Journal of Law, Medicine and Ethics*.^342^ Rivin et al determined that given the strong evidence for diverse benefits and very low risk, in the light of current international conventions it would be unethical not to recommend EIMC.^366^

Evaluation of US and international statutes as well as US case‐law, including of cases used by a lawyer to support his arguments against MC^69^, revealed no precedent for outlawing parent‐approved MC of minors.^342^, ^368^ A report by the Tasmanian Law Reform Commission^360^ was shown by legal, public health, and medical experts to be seriously flawed.^371^ If it was “*unlawful for physicians to circumcise*,”^69^ then EIMC would not be one of the most common medical procedures in the United States.^366^ It was noted, moreover, in a detailed treatise by a US lawyer opposed to MC that, “*Most circumcision lawsuits go nowhere*.”^384^

An evaluation pointed out that those who condemn parent‐approved MC of boys are not as quick to condemn other procedures in children, such as ear‐piercing, cosmetic orthodontia, surgery for correction of harelip and tongue‐tie, removal of supernumerary digits, and treatment of dwarfism by growth hormone injections.^323^ Removal of birthmarks and moles can be included. It was suggested that these interventions should be regarded by parents and physicians as being beneficial to the child, and that it seemed odd that infant MC is regarded by some as controversial.^323^

### 4.17 Logic

#### 4.17.1 False equivalence

It has been pointed out that, unlike EIMC, it is not the practice to routinely cut off ear lobes and breast buds to prevent future cancers or to remove the appendix to prevent appendicitis.^21^ The fallacy of false equivalence was invoked in disputing the argument.^385^, ^386^ It was pointed out that the breast is a body part with an important function. In contrast to MC, none of the other proposed prophylactic interventions would come close to the outcome of risk‐benefit (Table 9) or cost‐benefit analyses obtained for EIMC.

Another example we found was associating MC with female genital cutting/mutilation, the more extreme forms of which cause severe harm. The closest female equivalent of MC, clitoral hoodectomy, was introduced in the 1950s for women with an excessive or phimotic clitoral foreskin.^387^, ^388^ In a sexual dysfunction clinic in Boston, severity of clitoral phimosis was associated with increased likelihood of anorgasmia.^389^ We could find no recent evidence for clitoral hoodectomy to treat anorgasmia, but did find a recent study for treatment of severe clitoral phimosis and lichen sclerosus, that resulted in a significant increase in the patients’ Female Sexual Function Index Score.^390^ There is no scientific reason to equate the strong arguments favoring MC because of its multiple medical benefits with female genital mutilation or other genital procedures devoid of proven medical benefits, which would include labioplasty in high‐income countries to improve cosmesis.

#### 4.17.2 Genetic fallacy

Historical anecdotes, such as a belief by some in Victorian times that MC could be used to cure masturbation, have been used by opponents to dismiss MC.^391^ It has been suggested that irritation from balanitis, smegma, and infections could cause an uncircumcised boy to touch his penis, leading to stimulation and masturbation, behaviors frowned on in Victorian times.^392^, ^393^ A major 1913 textbook that expressed disdain for masturbation, made no mention of MC as a “cure.”^394^

MC is an ancient practice.^395^, ^396^ Evidence of MC in Europe during the Upper Paleolithic era (38 000‐11 000 BCE) was found in portable art and rock art at that time.^395^ It was suggested that the practice of MC may have accompanied the radiation of *Homo sapiens* out of Africa^396^ ∼220 000 years ago.^397^ It has further been suggested that privation and other forces explain why MC subsequently ceased in some cultures.^396^ In Victorian times, health benefits, such as protection against syphilis,^398^ balanitis, inferior hygiene, and phimosis,^399^, ^400^ have been used to explain why MC became popular in Anglophone countries.^396^ MC is common in diverse cultures globally.^401^ Ancient practices such as MC and hand‐washing may have stemmed from disease prevention measures. Over time these may have been subsumed into religious custom.^396^ The reasons humans might have had for MC hundreds or thousands of years ago can nevertheless be separated from the reasons for medical MC in contemporary society, the latter being based on sound scientific evidence described above, this being independent of earlier reasons.

### 4.18 Cost effectiveness

In the United States, a downturn in MC prevalence has been attributed to weak pediatric policy statements prior to 2012, increased immigration from countries in which MC is less common, a reduction in access and affordability, and lobbying by organizations opposed to MC.^316^ Similar trends occurred in Australia from the 1970s and the United Kingdom from the mid‐20th century. In the United States, this has included cessation of Medicaid coverage for elective MC in 18 States. US studies show that, in the long‐term, costs will be substantially higher because of the need for later, more expensive, medically indicated MC,^240^, ^402^, ^403^, ^404^, ^405^ which carries a 10‐ to 20‐fold higher risk of an adverse event,^51^ and for treatment of a wide array of conditions that EIMC protects against.^240^, ^370^, ^402^, ^403^, ^404^, ^405^, ^406^, ^407^ One study, of UTI and STIs in the United States, estimated that if MC declined from current levels to a level of 10%, costs would escalate to in excess of US$4.4 billion over 10 annual birth cohorts, the increase in expenditure being $313 per foregone MC.^402^ Just for HIV in the United States, the “*associated indirect costs may be more than 4 times the total direct medical expenses*.”^408^ It was suggested that if other conditions prevented by MC, as well as the indirect costs, were to be considered, the true cost would be considerably higher.^402^ For prostate cancer in the United States, in the absence of MC, it was estimated that there would be 24‐40% more cases and US$0.8‐1.1 billion extra in costs for treatment and terminal care per year.^355^ The CDC found MC in the United States was cost‐saving for HIV prevention in black and Hispanic males in whom HIV prevalence is highest.^240^

Medicaid noncoverage in several US states has made MC unaffordable for poor families. The ensuing decrease in infant MC has been estimated to result in > 100 additional HIV cases and $30 M in net medical costs for treatment per year.^403^ The cost to circumcise males in that birth cohort was US$4 856 000, that is, 6% of the cost of treating just HIV. Modelling studies have, moreover, found cost savings initially generated by noncoverage of elective infant MC by Medicaid in Louisiana^404^ and Florida^405^ were mitigated by increases in rate and expense of medically indicated MC. The Louisiana study only considered the costs of later MC for boys aged 0‐5 years. Lifetime costs would therefore represent a far greater financial burden on healthcare systems. The Florida study found Medicaid defunding led to a 6‐fold rise in publicly funded MCs at a cost of US$112 M,^405^ leading Florida to restore Medicaid coverage for nonmedical MC.^409^

Medical MC is enormously cost‐saving in high‐HIV settings,^410^ and continues to be rolled out in 14 high‐priority sub‐Saharan African countries with the approval of the WHO, the US CDC, UNAIDS, PEPFAR, the Bill & Melinda Gates Foundation, and local government and medical bodies dealing with the epidemic. Since EIMC is more cost‐effective, procedurally simpler, has a lower risk of adverse events, is quicker, more convenient, acceptable, and confers immediate benefits, albeit with a considerable lag before its HIV protection benefits begin, the CDC has recommended EIMC in 12 of these countries.^411^ VMMC may also be cost‐saving for MSM in China.^412^ Results from MC acceptability surveys have been summarized.^413^

### 4.19 US and non‐US policies

Affirmative MC statements arose from reviews by the AAP 2012^1^, ^2^ and CDC.^3^ Although the Canadian Pediatric Society (CPS) produced a position statement in 2015, it only recommended MC for males in high‐risk situations.^7^ Its recommendations stemmed from a faulty risk‐benefit analysis that was subsequently performed correctly by critics.^268^ The CPS responded to the criticisms,^63^ but their response was also seriously flawed.^414^

Current policies in other countries are negative and out‐of‐date. The 2010 Royal Australasian College of Physicians (RACP) policy^5^ led to a detailed critique by Fellows of the RACP and other medical bodies showing it was not evidence‐based.^415^ A defence by the chair of RACP committee^185^ was repudiated.^416^ Arguments by another commentator^417^ were also criticized.^418^ Guidance by the British Medical Association failed to involve a review of the medical literature and thus cannot be relied on.^4^ The only formal MC policy in Europe is by the Royal Dutch Medical Association.^6^ It was formulated by Gert van Dijk, a philosopher having no relevant scientific or medical background, and appears ideology‐based, rather than science‐based. Significantly, none of the policies opposing MC of boys denies the importance of MC in high‐HIV settings, or make extravagant claims about foreskin function.

In summary, our detailed evaluation of the high‐quality evidence shows that policies opposing MC are outdated and are not based on scholarly reviews of the medical scientific literature. At this point, we conclude that only policies by US medical bodies^1^, ^2^, ^3^ and the Circumcision Academy of Australia,^315^ each involving a detailed evaluation of the scientific evidence, should be relied on.

### 4.20 Consequences

Based on our evaluation of the scientific evidence, a downturn in MC, as intended by MC opponents, would have a detrimental impact on public health and individual wellbeing. This will in turn drive up costs for treatment of ensuing foreskin‐related conditions, and result in a rise in more expensive medical MC in older males.

Men circumcised as adults are able to compare their experience before and after MC, but men circumcised as infants have no experience to draw upon. Arguments used opposing MC can result in psychological problems^419^ and their sequelae^420^ in vulnerable men. The risk of distress, depression, and the broader psychological impact of arguments opposing MC in vulnerable men, and parents, merit further investigation.

In light of the above, one might appreciate the importance of MC education based on strong scientific evidence to help individuals evaluate contrary “evidence” used to oppose MC. Pseudoscience concerning HIV and AIDS led the then South African President, Thabo Mbeki, to disavow antiviral drugs, leading to loss of 330 000 lives and to 35 000 babies being born with HIV.^421^ More pervasive globally has been vaccine denialism. This has contributed to rises in measles, influenza, pertussis, polio, and other potentially fatal infections in Europe, the United States, Australia, and other countries. The Internet and social media outlets in particular facilitate the spread of disproven antivaccination information and arguments.^422^, ^423^ Deeper quantitative analysis has revealed that individuals opposing vaccination also tended to post material against other health‐related practices such as water fluoridation and MC.^424^ Denialism is also eroding efforts to ensure an effective response to anthropogenic climate change. Inadequate scientific literacy amongst some in society may be contributing to such extreme views.

### 4.21 Limitations

A limitation of this study is that many arguments opposing MC are absent from the scientific literature, but are popular on anti‐MC websites and social media. Searching only publication databases will miss these. We addressed this limitation to some extent by examining the *“16 functions of the foreskin”* meme (Table 3), which is particularly popular, as an Internet search will show. Some others are mentioned where they are relevant to published claims. But others will, inevitably, be overlooked as our review gave priority to published claims, these being the ones more likely to be influential to health care professionals. Not being in the peer‐reviewed scientific literature necessarily reduces the credibility of certain claims. It is to be hoped that health care professionals at least should be wary of claims that are not supported by scientific evidence published in reputable journals.

Another limitation is that the degree of benefit over the long‐term may be higher than evident from age‐restricted or short‐term studies. For example, early studies of UTIs in infancy found MC conferred a 10‐fold risk reduction, but only 1% of uncircumcised males were diagnosed with a UTI in the first year of life, whereas inclusion of data for older children and men found the ongoing risk reduction conferred by MC meant overall lifetime risk reduction was 4‐fold, but the proportion of uncircumcised males experiencing a UTI over their lifetime was 32% compared with 8.8% for circumcised males.^25^ Long‐term follow‐up of the three MC and VMMC RCTs have shown a continuation of level of effectiveness of approximately 60% in two of these,^203^, ^425^ and an increase in effectiveness in another RCT to 73%.^426^ Thus, with larger and wider studies we expect the data will continue to consolidate and may show an increase in the strength of the protective effect conferred by MC.

The specific focus of our evaluation is another potential limitation. The purpose of our systematic review was to assess the scientific and medical data, including data on sexual function. We did not address psychosocial, religious, or emotional arguments that might be posed. Nor did we address local or regional factors, MC practice in developed countries versus developing countries, or Muslims versus others.

## 5 CONCLUSIONS AND IMPLICATIONS

The present systematic review has contrasted evidence used to argue against MC with evidence from RCTs, systematic reviews, and meta‐analyses, in particular, that has demonstrated the multiple medical and health benefits and low risk of MC to males^269^, ^316^, ^427^ and their female sexual partners.^249^, ^303^ The key publications forming the framework of the present systematic review are provided in Table 10, as required by PRISMA guidelines. We find that, based on the evidence rated by quality, MC, especially when performed in early infancy, is favored.

**Table 10:** PRISMA‐required summary of the key publications on each topic cited in this systematic review

Topic	
Data and arguments opposing MC	Critique(s) of each respective article
*Urinary tract infections*	
Singh‐Grewal et al 2005^33^	Schoen 2005,^35^ Morris & Wiswell 2013^25^
Van Howe 2005^34^	Simforoosh et al 2012^36^
*Deaths from infant MC*	
Bollinger 2010^55^	Morris et al 2012^56^
*Meatal stenosis*	
Van Howe 2006^73^	Schoen 2007^74^
Frisch & Simonsen 2018^71^	Morris & Krieger 2017,^78^ Morris & Krieger 2018^72^
Van Howe 2018^75^	Morris & Krieger 2018^77^
*Alexithymia*	
Bollinger & Van Howe 2011^108^	Morris et al 2012^111^
*Anesthesia*	
Paix 2012^95^	Dilley & Morris 2012^97^
*Autism spectrum disorder*	
Frisch & Simonsen 2015^93^	Bauer 2015,^101^ Morris & Wiswell 2015,^94^ Sneppen & Thorup 2016^102^
*Sexual function and pleasure*	
O'Hara & O'Hara 1999^131^	Cortéz‐González et al 2008,^146^ Kigozi et al 2009^149^
	Zulu et al 2015^148^
Boyle & Bensley 200^132^	Morris & Krieger 2013^119^
Kim & Pang 2007^133^	Willcourt 2007^138^
Sorrells et al 2007^134^	Waskett & Morris 2007,^139^ Morris & Krieger 2013,^119^ Cox et al 2015,^143^ Bossio et al 2016^144^
Frisch et al 2011^135^	Morris et al 2012,^140^ Morris et al 2013^119^
Bronselaer et al 2013^136^	Morris et al 2013^141^
Boyle 2015^137^	Morris & Krieger 2015^142^
*HIV*	
Van Howe 1999^175^	Moses et al 1999^207^
	O'Farrell & Egger 2000^208^
Green et al 2008^176^	Wamai et al 2008^209^
Gisselquist et al 2009 177	Wamai et al 2011^112^
Green et al 2010^178^	Banerjee et al 2011^210^
Boyle & Hill 2011^179^	Wamai et al 2012^115^
Boyle & Hill 2011^180^,	Cooper et al 2011,^222^ Morris et al 2012^56^
Chin 2011,^181^ Conroy 2011,^182^	Cooper et al 2011,^222^ Morris et al 2012^56^
Darby 2011,^183^ Darby &	Cooper et al 2011,^222^ Morris et al 2012^56^
Van Howe 2011,^184^ Forbes 2011,^185^	Cooper et al 2011,^222^ Morris et al 2012^56^
Paix 2011,^186^ Travis et al 2011^187^	Cooper et al 2011,^222^ Morris et al 2012^56^
Van Howe & Storms 2011^188^	Morris et al 2011^211^
de Camargo et al 2013^189^	Wamai et al 2015^217^
de Camargo et al 2015^190^	Wamai et al 2015^218^
Van Howe 2015^191^	Morris et al 2018^220^
Van Howe 2018^192^	Morris et al 2017^221^
*Other STIs*	
Van Howe 2007^257^	Castellsague et al 2007^259^
Van Howe 2007^256^	Waskett et al 2009^260^
Van Howe 2009^258^	Morris et al 2014^255^
Van Howe 2013^257^	Morris et al 2014^255^
Darby 2015^290^	Morris et al 2017^219^
*MC can be delayed (“self‐determination”)*	
Darby 2013^18^	Morris et al 2012^312^
Merkel & Putzke 2013^319^	Morris et al 2012^312^
Darby 2015^320^	Morris et al 2012^312^
Van Howe 2015^310^	Morris et al 2012^312^
*Treatment of inflammatory conditions*	
Frisch & Earp 2018^329^	Morris & Krieger 2017,^330^ Folaranmi et al 2018^331^
*Penile cancer*	
Preston 1970^338^	Dagher et al 1973^340^
Van Howe & Hodges 2008^339^	Waskett & Morris 2008^341^
Svoboda et al 2016^26^	Morris et al 2017^342^
*Legal, ethical*	
Green et al 2009^359^	Leibowitz et al 2009^369^
	Morris et al 2009^370^
Tasmanian Law Reform Institute 2010^360^	Bates et al 2013^371^
Hill et al 2012^361^	Bates & Morris 2012^372^
Svoboda 2014^362^	Morris 2014^373^
Darby 2015^320^	Morris et al 2016^367^
Adler 2016^69^	Rivin et al 2016^366^
Svoboda et al 2016^26^	Morris et al 2017^374^
*2012 AAP policy on EIMC*	
Frisch et al 2013^357^	AAP Task Force 2013^363^
Svoboda & Van Howe 2013^311^	Morris et al 2014^364^
Jenkins 2014^375^	Morris et al 2014^377^
Darby 2014^376^	Morris 2014^378^
Darby 2015^320^	Morris et al 2016^367^
Svoboda et al 2016^26^	Brady 2016^379^
	Morris et al 2017^374^
*2014 CDC MC draft policy*	
Earp 2015^358^	Morris 2015^365^
Adler 2016^69^	Rivin et al 2016^366^
Frisch & Earp 2018^329^	Morris et al 2017^368^
	CDC^380^
*2010 RACP policy on EIMC*	
RACP 2010^5^	Morris et al 2012^415^
Forbes 2012^185^	Morris et al 2012^416^
Jansen 2016^417^	Wodak et al 2017^418^
*2015 CPS policy on EIMC*	
Sorokan et al 2015^7^	Morris et al 2016^268^
Robinson et al 2017^63^	Morris et al 2017^414^

One should be aware of confirmation bias and asymmetric Bayesianism^428^ when it comes to any discussion of a contentious topic such as MC. A recent study revealed, moreover, that *ad hominen* attacks on scientists themselves, rather than the empirical basis of the science, are an effective strategy by those who reject scientific evidence on a topic.^429^

Following the 2012 AAP infant MC policy, a commentary in *AAP News* 430 suggested that the statement by the AAP Task Force that, *“It is important that clinicians routinely inform parents of the health benefits and risks of male newborn circumcision in an unbiased and accurate manner,”* may require pediatricians, at least those in the United States, to modify their discussions about newborn health interventions with parents, since, “*physicians sometimes can be held accountable for harm that results from not telling patients about an available medical treatment or procedure*.”^430^

The AAP suggested that after evaluation of the evidence by parents those individuals should be free to either consent to having their son circumcised or decline MC for a son.^430^ Women can have considerable power in regard to the decision. They can influence the choice of EIMC or later MC for their sons,^312^ brothers, other male family members, and friends. They can, moreover, choose to have a circumcised sexual partner, or encourage an uncircumcised partner to undergo the procedure.

The present systematic review should help prioritize the best scientific evidence when it comes to MC, especially EIMC, as an important public health issue worldwide. It should also provide a useful resource for those confronted with contrary information.

## CONFLICTS OF INTEREST

The first author is a member of the Circumcision Academy of Australia, a not‐for‐profit, government registered, medical association that provides evidence‐based information on male circumcision to parents, practitioners and others, as well as contact details of doctors who perform the procedure. The second author is an editor for http://CircFacts.org. The third author provided advice and supported the legal help to University of Washington for the patenting of a circumcision device. He did not receive any income from this. The authors have no religious or other affiliations that might influence the topic of MC.

## Supporting information

Supporting Information
