# Supplementary material for: Critical evaluation of arguments opposing male circumcision: A systematic review
Source: J Evid Based Med. 2019 Sep 8;12(4):263–90. doi: 10.1111/jebm.12361 (PMC6899915; doi:10.1111/jebm.12361)
Supplement: Supplementary file 1 — Supporting Information [file JEBM-12-263-s001.docx]

SUPPLEMENTARY INFORMATION

PubMed search results from 1 January 2005 through 31 December 2018, showing number of “hits” each year using as keyword "circumcision", together with the additional keyword shown in left-hand column.

**Keyword 2005 2006 2007 2008 2009 2010 2011 2012 2013 2014 2015 …cont.**

Adverse events 4 3 9 7 13 14 19 24 17 24 18

Bacterial vaginosis 2 0 0 2 1 6 1 7 5 3 4

Balanitis 10 11 13 12 12 10 11 16 13 7 4

Balanoposthitis 2 3 0 1 0 3 4 7 2 0 2

Best time 1 4 2 1 1 0 2 5 1 1 1

Cervical cancer 1 8 5 0 6 8 7 16 14 6 5

Chancroid 1 2 4 0 0 2 0 2 1 1 6

Chlamydia 3 9 6 4 3 8 2 4 2 2 3

Complications 66 84 76 86 84 101 83 108 121 90 82

Cost effectiveness 6 7 7 10 13 20 11 16 12 16 10

HIV 63 92 182 159 151 172 184 191 171 147 146

HPV 5 10 8 7 17 23 22 30 22 12 14

HSV-2 6 6 12 5 20 15 15 10 6 6 5

Human

papillomavirus 6 12 8 8 23 29 23 32 26 10 10

Penis cancer 11 28 16 15 18 15 25 21 17 7 12

Hygiene 8 12 18 11 13 16 15 23 13 14 19

Lichen sclerosus/is 5 1 2 4 7 4 11 4 7 3 0

Microbiome 0 0 0 0 0 2 2 4 1 1 1

Mycoplasma 0 1 0 0 0 2 0 3 1 1 1

Paraphimosis 4 3 3 5 2 4 4 7 3 0 2

Penile cancer 14 32 17 17 21 20 27 29 24 7 16

Phimosis 29 36 27 34 33 28 28 37 39 20 18

Pleasure 3 3 2 6 9 5 3 10 9 3 9

Policy 16 23 38 23 27 38 42 52 39 40 51

Prostate cancer 2 2 4 1 4 0 0 5 4 3 1

Public health 162 187 240 237 248 266 258 295 277 195 189

Risk-benefit 17 13 23 23 27 33 20 33 27 19 32

Satisfaction 15 12 19 23 20 25 24 29 29 22 18

Sensation 9 7 11 4 6 8 3 9 15 9 8

Sensitivity 7 13 10 9 11 9 11 12 13 5 3

Sexual function 12 14 22 20 20 31 28 34 27 20 19

Sexually trans-

mitted infection 67 100 174 147 135 163 162 174 152 90 95

Syphilis 3 5 14 2 8 8 7 6 4 10 10

Timing 4 0 2 3 3 2 3 2 5 3 0

Trichomonas 1 0 2 4 3 5 0 4 2 0 0

Urinary tract

infection 13 17 13 11 13 19 13 29 23 11 16

------------------------------------------------------------------------------------------------------------------

… continued on next page

… continued:

**Keyword 2016 2017 2018 Total**

Adverse events 20 18 11 201

Bacterial vaginosis 0 1 0 32

Balanitis 9 14 13 155

Balanoposthitis 1 2 3 30

Best time 2 3 3 27

Cervical cancer 6 5 1 88

Chancroid 0 0 0 19

Chlamydia 2 4 1 47

Complications 76 67 56 1180

Cost effectiveness 25 12 5 170

HIV 147 87 75 1951

HPV 8 10 10 199

HSV-2 2 2 2 113

Human

papillomavirus 7 12 5 212

Hygiene 16 19 9 206

Lichen sclerosus 4 10 8 70

Microbiome 1 2 0 14

Mycoplasma 0 0 0 9

Paraphimosis 1 1 2 418

Penile cancer 6 11 12 214

Phimosis 25 27 18 596

Pleasure 4 6 2 74

Policy 42 26 19 476

Prostate cancer 5 3 0 34

Public health 234 164 107 3039

Risk-benefit 29 20 12 328

Satisfaction 33 23 17 309

Sensation 7 11 4 111

Sensitivity 14 6 6 129

Sexual function 16 21 15 299

Sexually trans-

mitted infection 96 62 28 1645

Syphilis 3 3 1 84

Timing 6 5 4 42

Trichomonas 0 3 0 24

Urinary tract

infection 14 12 5 209

------------------------------------------------------------------------------------------------------------------

Grand total = 12,754 “hits”*

*The numbers shown do not take into account duplication of publication in different searches.
